# The benefits evaluation of abdominal deep inspiration breath hold based on knowledge‐based radiotherapy treatment planning for left‐sided breast cancer

**DOI:** 10.1002/acm2.13013

**Published:** 2020-09-12

**Authors:** Jiaqi Xu, Jiazhou Wang, Feng Zhao, Weigang Hu, Guorong Yao, Zhongjie Lu, Senxiang Yan

**Affiliations:** ^1^ Department of Radiation Oncology The First Affiliated Hospital College of Medicine Zhejiang University Hangzhou Zhejiang China; ^2^ Department of Radiation Oncology Fudan University Shanghai Cancer Center Shanghai China

**Keywords:** breast cancer, deep inspiration breath hold, dose distribution prediction, knowledge‐based planning, machine learning

## Abstract

**Purpose:**

To study the impact of abdominal deep inspiration breath hold (DIBH) technique on knowledge‐based radiotherapy treatment planning for left‐sided breast cancer to guide the application of DIBH technology.

**Materials and methods:**

Two kernel density estimation (KDE) models were developed based on 40 left‐sided breast cancer patients with two CT acquisitions of free breathing (FB‐CT) and DIBH (DIBH‐CT). Each KDE model was used to predict dose volume histograms (DVHs) based on DIBH‐CT and FB‐CT for another 10 new patients similar to our training datasets. The predicted DVHs were taken as a substitute for dose constraints and objective functions in the Eclipse treatment planning system, with the same requirements for the planning target volume (PTV). The mean doses to the heart, the left anterior descending coronary artery (LADCA) and the ipsilateral lung were evaluated and compared using the T‐test among clinical plans, KDE predictions, and KDE plans.

**Results:**

Our study demonstrated that the KDE model can generate deliverable simulations equivalent to clinically applicable plans. The T‐test was applied to test the consistency hypothesis on another ten left‐sided breast cancer patients. In cases of the same breathing status, there was no statistically significant difference between the predicted and the clinical plans for all clinically relevant DVH indices (*P* > 0.05), and all predicted DVHs can be transferred into deliverable plans. For DIBH‐CT images, significant differences were observed between FB model predictions and clinical plans (*P* < 0.05). DIBH model prediction cannot be optimized to a deliverable plan based on FB‐CT, with a counsel of perfection.

**Conclusion:**

KDE models can predict DVHs well for the same breathing conditions but degrade with different breathing conditions. The benefits of DIBH for a given patient can be evaluated with a quick comparison of prediction results of the two models before treatment planning.

## 1 BACKGROUND

Postoperative adjuvant radiotherapy (RT) plays an indispensable role in breast‐conserving treatment to minimize the risks of local‐regional recurrence and metastasis. Whole breast irradiation (WBI) after breast‐conserving surgery as a comprehensive treatment model, which has been confirmed to possess the similar local control and overall survival rates to modified radical surgery in breast cancer patients.^1^ However, the dose of the surrounding critical organs‐at‐risks (OARs), especially the heart, left lung and the left anterior descending coronary artery (LADCA),^2^, ^3^, ^4^ are crucial to the RT quality assessment for left‐sided breast cancer.

Therefore, by using a diversity of methods, such as DIBH, intensity modulated radiation therapy (IMRT) techniques, treatment in the prone position and proton therapy, to shield the heart and minimize the lung and LADCA doses while ensuring enough dose in the target volume during left‐breast postoperative radiotherapy have been presented. Comparing DIBH and IMRT, IMRT is the most commonly used strategy in left‐sided breast postoperative radiotherapy.^5^, ^6^, ^7^ The DIBH maneuver we used is the abdominal DIBH (A‐DIBH), which could widen the spatial Euclidean distances between the heart and the target volume. IMRT treatment technique has the capability of reducing the cardiac dose while delivering adequate target coverage because of its unique dose calculation and beam weight optimization.

The selection of the final radiotherapy regimen (especially the selection of respiratory mode) will greatly affect the normal tissue complications (NTCP) and tumor control rate.^8^, ^9^ In the previous IMRT plans, physicians often determined the ideal OAR dose volume limit through population‐based recommendations (either from the tumor radiotherapy team or from the doctor's intuition).^10^ However, the huge geometric differences in the complexity of PTV and OAR among patients make it a challenge for doctors to quickly and accurately select the best ultimate treatment for a particular patient within all acceptable options.

Knowledge‐based planning (KBP) is a promising technology. There is a large amount of image information and dose planning information of cancer patients in the current radiotherapy system, which has become a priori knowledge. By feature extraction and quantitative analysis of these prior knowledge, a reliable empirical model (KBMs) can be obtained to realize the intelligence of the radiotherapy planning system. Current studies have proved that KBP has a higher consistency of plan quality and higher operational efficiency than manual plans with different quality.^11^, ^12^, ^13^ For example, RapidPlan™(Varian Medical Systems, Palo Alto, CA, USA) has been widely used as a commercial KBP product.^11^, ^14^

In the KBP method, the prediction of DVH in new patients requires the use of the DVH of OAR in the previous clinical plan and the parameterized model generated by the relevant anatomical structure,^13^, ^15^ thus emphasizing the importance of the parameterized prior model. However, it remains to be seen whether the implementation effect of the parameterized prior model in KBP is consistent under different breathing conditions. To our best knowledge, the impacts of different breathing methods during CT simulation for left‐sided breast cancer on knowledge‐based treatment planning have not been reported before.

Therefore, this study established two knowledge‐based empirical models for the treatment of the same group of breast cancer patients based on different respiratory conditions. We then used these two KBMs to cross‐predict CT in both breathing patterns, creating four KBP plans for each patient. We attempted to investigate the compatibility of KBP with different respiratory conditions, such as whether the DIBH KBM is applicable to FB‐CT prediction, or whether the FB KBM is applicable to DIBH‐CT prediction. Quantifying the benefits of using the specified model can help us clearly understand the use of KBM to predict the OAR dose of postoperative radiotherapy for breast cancer and guide the application of A‐DIBH radiotherapy technology.

## 2 Method and materials

**Fig. 1:**
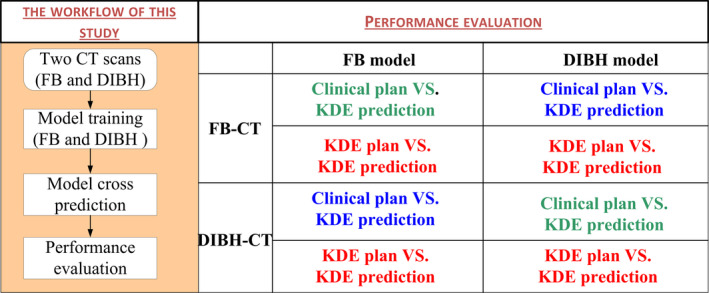
The strategy of knowledge‐based planning generated optimization objectives.

The workflow of this study is illustrated in the diagram in Fig. 1. Firstly, two KBMs (FB model and DIBH model) were built from 40 existing clinical plans that have ideal tumor coverage and OAR doses with different breath settings. Another ten new patients were selected to investigate the performance of the prediction model. Two treatment plans were established with uniform standards by the experienced physicist, and then confirmed by another senior physicist.

DVHs of these ten new patients were estimated by two KBMs. The estimated DVHs were taken as a substitute to dose constraints and objective functions in Eclipse treatment planning system (TPS) for each new patient.

There are three types of comparisons we want to investigate in this study. Firstly, we wanted to confirm whether our model could precisely predict the DVHs with same breath settings, such as using FB model to predict patients with FB‐CT. These comparisons were marked “green” in Fig. 1.

Secondly, we wanted to investigate whether the model built with one breath condition can precisely predict DVH for patients with another breath. Such as using FB model to predict the DVH for the patient with DIBH‐CT images. These comparisons were marked “blue” in Fig. 1.

Third, we wanted to investigate whether these DVH prediction models can be optimized to a deliverable plan. These comparisons were marked “red” in Fig. 1.

### 2.A Patients and treatment planning

The training dataset consisted of 40 consecutive patients who received adjuvant radiotherapy after breast conserving surgery for left breast cancer. The mean age of those patients was 45.7 (range, 28–70), and the median age was 49. Each patient underwent two CT simulation scans with a Siemens Sensation Open 24‐slice scanner (Siemens, Forchheim, Germany): the FB condition and the A‐DIBH condition. For consistency, all patients were practiced A‐DIBH according to audio and visual coaching for at least a week before the simulation scan, until they can repeat the mode and hold their breath for 15–20 s with the auxiliary of Varian Real‐time Position Management (RPM) System.

To achieve optimal homogeneity of the data in the present analysis, we incorporated only the whole‐breast irradiation series. Target volumes and OAR were entirely contoured via two CT series in the Eclipse treatment planning system (Varian Medical Systems, Palo Alto, CA, USA) according to the Danish Breast Cancer Cooperative Group (DBCG) atlas.^16^ Two intensity modulated radiotherapy treatment plans were generated in the Eclipse for each CT, using the anisotropic analytical algorithm (AAA) for final dose calculation. All IMRT plans containing 6 fixed non‐opposing fields, and thegantry angles and beam energies of each plan are the same as the clinical methods.

The criterion of treatment plans was that 97% of the PTV should be covered by at least 95% of the isodose (and <108% of the isodose), and the mean dose of PTV in the whole cases was prescribed to 50 Gy in 25 fractions.

### 2.B KDE model training

Inspired by Skarpman's KDE algorithm,^15^ the two‐parameters KDE which incorporated two predictive features was implemented to predict DVHs. It calculates the conditional probability density of the dose *d* given *x* (the signed minimal distance between the voxels on the PTV surface and in the OAR) and *θ* (the angle between *x* and the center of the CT image) from the training dataset.

Two KDE prediction models were developed based on 40 left‐sided breast cancer patients with two different CT scans of FB and DIBH in the aforementioned high‐quality IMRT cases. These cases were planned by the experienced dosimetric and approved for clinical treatment by attending physicians. Each KBM was applied to its training dataset then. The estimated DVH was compared with the clinical DVH to verify the reliability of the KDE model.

### 2.C DVH prediction and plan optimization

Another ten patients similar to our training datasets were enrolled for different model evaluations. Each patient has two images, FB‐CT and DIBH‐CT. The DVHs of each image was estimated by two KDE models, FB and DIBH model. So, each patient has four estimated DVHs, marked as KDE predictions.

To demonstrate whether these estimated DVHs can be directly used to generate deliverable plans, we created another four plans based on four cross‐estimated DVHs. We use the estimated DVHs generated in the previous step as dose constraints and objective functions at the specific points without any additional auxiliary human intervention (Table 1). The KDE plan was optimized on the Eclipse treatment planning system then. The gantry angles of these plans were exactly the same as the original clinical plans.

**Table 1:** OAR dose constraint points used for plan optimization for 200 cGy/fx plan in 25 fractions.

Organ	Dose constraints points
Heart	D2% D_mean_ V5 V10 V20 V30
LAD	D2% D_mean_ V5 V10 V20 V30
Lung	D2% D_mean_ V5 V10 V20 V30
Spinal Cord	D_max_ D_mean_
PTV	D_max_ D_mean_ D_min_

Each new patient has four KDE predictions, four KDE plans, and two clinical plans. Here we use superscript to identity plan type and use a subscript to identify the image. For example, the original clinical plans were marked as $Plan_{FB‐CT}^{\mathrm{manual}}$ and $Plan_{DIBH‐CT}^{\mathrm{manual}}$, KDE plans based on FB‐CT were marked as $Plan_{FB‐CT}^{FB\;\mathrm{mod}\;el}$ and $Plan_{FB‐CT}^{DIBH\;\mathrm{mod}\;el}$ for two KBMs, plans based on DIBH‐CT were marked as $Plan_{DIBH‐CT}^{FB\;\mathrm{mod}\;el}$ and $Plan_{DIBH‐CT}^{DIBH\;\mathrm{mod}\;el}$ for two KBMs. It is noticeable that in all IMRT plans, the PTV requirements were the same.

### 2.D Dosimetric comparison

A paired student's *T*‐test was used to assess the significance of any differences in dose metrics where significance corresponded to a *P*‐value 0.05. Mean doses to the heart, left anterior descending coronary artery (LADCA), and left lung were compared.

## 3 RESULTS

### 3.A The performance of the KDE models

The results (Table 2) show that there were no differences between clinical plans and KDE predictions for both models in the training dataset, confirming that the DVH estimation of the KBM was successful.

**Table 2:** Summary of organs‐at‐risk (OAR) doses from intensity‐modulated radiation therapy (IMRT) validation, comparing the clinical plan and dose volume histogram (DVH) estimates from its KBM for 40 cases (mean ± SD).

Structure	CT	Parameters	Clinical plan (Gy)	KDE prediction (Gy)	Clinical plan vs KDE prediction (*P*‐value)
Heart	FB	Mean (Gy)	1.96 ± 0.26	1.95 ± 0.34	0.77
		V5 (%)	6.51 ± 3.34	6.66 ± 1.64	0.91
		V20 (%)	0.57 ± 0.55	0.69 ± 0.99	0.74
	DIBH	Mean (Gy)	1.30 ± 0.31	1.31 ± 0.23	0.87
		V5 (%)	3.25 ± 1.04	3.47 ± 2.33	0.76
		V20 (%)	0.21 ± 0.21	0.13 ± 0.39	0.51
LADCA	FB	Mean (Gy)	16.57 ± 0.33	16.02 ± 0.28	0.48
		V5 (%)	81.84 ± 6.91	80.16 ± 6.02	0.52
		V20 (%)	36.18 ± 11.67	34.51 ± 16.82	0.80
	DIBH	Mean (Gy)	8.59 ± 3.73	8.47 ± 3.08	0.76
		V5 (%)	49.09 ± 9.30	46.86 ± 14.79	0.70
		V20 (%)	11.98 ± 13.34	11.81 ± 10.12	0.97
Left lung	FB	Mean (Gy)	5.53 ± 1.42	5.48 ± 1.15	0.48
		V5 (%)	19.73 ± 2.72	19.03 ± 2.32	0.49
		V20 (%)	9.64 ± 1.84	9.40 ± 1.60	0.71
	DIBH	Mean (Gy)	5.43 ± 0.59	5.48 ± 0.60	0.78
		V5 (%)	19.85 ± 2.06	20.03 ± 2.94	0.85
		V20 (%)	9.23 ± 1.28	9.28 ± 1.84	0.93

Note

Clinical plan represents the plan used in the model training dataset.

### 3.B The models work in same breath settings

The results of model performance in the same breath settings for another 10 left‐breast patients are presented in Table 3. There was no difference between clinical plan and estimated plan for all structures’ mean dose (*P* > 0.05). Meanwhile, all estimated DVHs can be transferred into deliverable KDE plans. No difference between prediction and KDE plan was observed (*P* > 0.05).

**Table 3:** The dose comparison of the OARs among the clinical plans, generated plans and the predicted DVHs performed on the same breath settings (mean ± SD)

Structure	CT	Parameters	Clinical plan	KDE prediction	KDE plan	Clinical plan vs prediction (*P*‐value)	prediction vs KDE plan (*P*‐value)
Heart	FB	Mean (Gy)	2.03 ± 0.38	2.00 ± 0.37	2.04 ± 0.38	N.S.	N.S.
		D2% (Gy)	16.04 ± 5.23	15.91 ± 5.08	15.97 ± 5.19	N.S.	N.S.
		V5 (%)	6.44 ± 2.62	6.37 ± 3.20	6.48 ± 3.16	N.S.	N.S.
		V20 (%)	1.01 ± 0.74	0.95 ± 0.62	0.98 ± 0.67	N.S.	N.S.
	DIBH	Mean (Gy)	1.17 ± 0.31	1.27 ± 0.21	1.29 ± 0.21	N.S.	N.S.
		D2% (Gy)	8.52 ± 3.05	8.62 ± 3.41	8.57 ± 3.34	N.S.	N.S.
		V5 (%)	2.86 ± 1.86	2.76 ± 1.74	2.83 ± 1.79	N.S.	N.S.
		V20 (%)	0.13 ± 0.21	0.15 ± 0.27	0.16 ± 0.26	N.S.	N.S.
LADCA	FB	Mean (Gy)	17.46 ± 5.01	16.01 ± 1.08	16.00 ± 1.20	N.S.	N.S.
		D2% (Gy)	29.75 ± 12.1	28.69 ± 8.11	28.32 ± 8.14	N.S.	N.S.
		V5 (%)	81.62 ± 14.8	79.87 ± 10.5	78.31 ± 10.6	N.S.	N.S.
		V20 (%)	35.98 ± 15.2	35.19 ± 20.8	35.41 ± 17.1	N.S.	N.S.
	DIBH	Mean (Gy)	6.70 ± 4.78	7.66 ± 1.60	7.79 ± 1.70	N.S.	N.S.
		D2% (Gy)	16.54 ± 6.91	17.61 ± 5.34	17.66 ± 5.58	N.S.	N.S.
		V5 (%)	44.02 ± 12.7	44.85 ± 8.34	44.39 ± 9.81	N.S.	N.S.
		V20 (%)	9.30 ± 11.51	8.05 ± 3.14	7.96 ± 4.09	N.S.	N.S.
Left lung	FB	Mean (Gy)	4.73 ± 1.62	5.41 ± 0.90	5.44 ± 0.99	N.S.	N.S.
		D2% (Gy)	29.33 ± 6.63	32.67 ± 7.14	33.74 ± 8.36	N.S.	N.S.
		V5 (%)	18.5 ± 4.67	19.95 ± 5.81	20.52 ± 4.14	N.S.	N.S.
		V20 (%)	8.17 ± 4.24	7.83 ± 3.37	7.82 ± 2.45	N.S.	N.S.
	DIBH	Mean (Gy)	4.88 ± 1.05	5.48 ± 0.59	4.76 ± 0.64	N.S.	N.S.
		D2% (Gy)	39.15 ± 2.37	39.23 ± 1.72	38.08 ± 1.46	N.S.	N.S.
		V5 (%)	19.53 ± 3.42	20.62 ± 2.33	20.31 ± 2.07	N.S.	N.S.
		V20 (%)	5.66 ± 2.83	5.77 ± 2.09	5.18 ± 1.57	N.S.	N.S.

Note

*<0.05,**<0.01,***<0.001, N.S.: not significant.

### 3.C The FB model works with DIBH‐CT

The result of the FB model works with DIBH‐CT was presented in Table 4. The mean dose of which the FB model predicted was higher than the clinical plan (*P* < 0.05 for all three structures). By transferring to deliverable KDE plan, the dose of the left lung and the V5 of the LADCA were reduced significantly (*P* < 0.05).

**Table 4:** The results of the free breathing (FB) model performed on deep inspiration breath hold‐computed tomography (DIBH‐CT) in 10 new left‐breast intensity‐modulated radiation therapy (IMRT) plans (mean ± SD).

Structure	Parameters	Clinical plan	KDE prediction	KDE plan	Clinical plan vs KDE prediction (*P*‐value)	KDE prediction vs KDE plan (*P*‐value)
Heart	Mean (Gy)	1.17 ± 0.31	1.42 ± 0.26	1.40 ± 0.26	*	N.S.
	D2% (Gy)	8.52 ± 3.05	8.69 ± 3.70	8.55 ± 3.11	*	N.S.
	V5 (%)	2.86 ± 1.86	3.08 ± 1.54	3.07 ± 1.32	*	N.S.
	V20 (%)	0.13 ± 0.21	0.19 ± 0.32	0.24 ± 0.21	*	N.S.
LADCA	Mean (Gy)	6.70 ± 4.78	12.1 ± 1.61	11.98 ± 1.51	**	N.S.
	D2% (Gy)	16.54 ± 6.91	27.50 ± 4.43	26.12 ± 8.53	**	N.S.
	V5 (%)	44.02 ± 12.7	62.40 ± 6.91	59.18 ± 6.74	***	*
	V20 (%)	9.30 ± 11.51	14.83 ± 6.02	14.52 ± 5.43	**	N.S.
Left lung	Mean (Gy)	4.88 ± 1.05	5.61 ± 1.08	5.24 ± 0.58	***	*
	D2% (Gy)	39.15 ± 2.37	42.85 ± 4.21	40.70 ± 4.96	**	*
	V5 (%)	19.53 ± 3.42	21.50 ± 3.91	20.95 ± 2.57	*	*
	V20 (%)	5.66 ± 2.83	8.65 ± 1.31	7.57 ± 2.45	*	*

Note

*<0.05,**<0.01,***<0.001, N.S.: not significant.

### 3.D The DIBH model works with FB‐CT

Table 5 shows the result of the DIBH model works with FB‐CT. Compared to the clinical manual plans, the KDE prediction resulted in lower mean doses of the heart and LADCA by 0.24 ± 0.36 Gy (*P* = 0.02), and 4.57 ± 2.46 Gy (*P* = 0.014), respectively. The left lung mean dose of the KDE prediction was 0.33 ± 0.99 Gy higher than the clinical plan (*P* = 0.01).

**Table 5:** The results of the deep inspiration breath hold (DIBH) model performed on free breathing‐computed tomography (FB‐CT) in ten new left‐breast intensity‐modulated radiation therapy (IMRT) plans (mean ± SD).

Structure	Parameters	Clinical plan		KDE prediction	KDE plan	Clinical plan vs KDE prediction (*P*‐value)	KDE prediction vs KDE plan (*P*‐value)
Heart	Mean (Gy)		2.03 ± 0.38	1.79 ± 0.31	2.09 ± 0.35	*	*
	D2% (Gy)		16.04 ± 5.23	12.45 ± 3.45	15.89 ± 5.45	*	*
	V5 (%)		6.44 ± 2.62	4.58 ± 1.47	6.49 ± 2.94	*	*
	V20 (%)		1.01 ± 0.74	0.46 ± 0.26	1.11 ± 0.78	*	*
LADCA	Mean (Gy)		17.46 ± 5.01	12.89 ± 1.85	17.86 ± 4.09	*	**
	D2% (Gy)		29.75 ± 12.1	34.71 ± 2.87	29.74 ± 12.9	*	*
	V5 (%)		81.62 ± 14.8	62.80 ± 4.27	88.93 ± 13.0	**	**
	V20 (%)		35.98 ± 15.2	23.26 ± 7.66	43.62 ± 27.1	*	**
Left lung	Mean (Gy)		4.73 ± 1.62	5.06 ± 1.01	5.69 ± 1.50	*	*
	D2% (Gy)		29.33 ± 6.63	37.22 ± 2.34	47.01 ± 1.96	*	*
	V5 (%)		18.5 ± 4.67	21.96 ± 3.65	18.36 ± 2.18	*	*
	V20 (%)		8.17 ± 4.24	9.17 ± 2.15	7.69 ± 2.03	*	*

Note

*<0.05,**<0.01,***<0.001, N.S.: not significant.

Significant differences were observed in all structures between the KDE plan and KDE prediction (*P* < 0.05 for all three structures). These predicted DVHs may not be directly transferred to a deliverable plan.

## 4 DISCUSSION

Deep inspiration breath hold offers increased lung volume and suppressed respiratory motion. As Schönecker et al.^17^ mentioned, DIBH could significantly reduce high dose areas and mean doses to the heart. Our study also proves the earlier results that abdominal deep inspiration breath hold is of great significance in protecting OARs during radiation for left‐sided breast cancer treatment. However, DIBH treatments may introduce more setup uncertainties such as unsuccessful guidance, resulting in more resource‐intensiveness than FB treatments.

It is important to determine early on how much a patient will benefit from DIBH. This research sought to reveal the impact of abdominal breath holding on knowledge‐based treatment planning for breast cancer radiotherapy so that we can guide the application of DIBH more precise before treatment.

In this study, two KDE‐based dose prediction models with two different respiratory patterns of FB and DIBH for IMRT treatment were established. The contrast of both FB and DIBH IMRT plans in the original 40 patients, created by the manual and KBP methods, shows that KBP plans provided at least comparable plan quality compared to clinical ones (*P* > 0.05).

A further comparative study was performed in another ten patients similar to the training datasets. There was no significant difference between $Plan_{FB‐CT}^{FB\;\mathrm{mod}\;el}$ and $Plan_{FB‐CT}^{\mathrm{manual}}$ or $Plan_{DIBH‐CT}^{DIBH\;\mathrm{mod}\;el}$ and $Plan_{DIBH‐CT}^{\mathrm{manual}}$. The acceptable *P*‐values indicate that our model provides good estimates for DVHs in the same breath settings. A counsel of perfection of ${\text{Prediction}}_{FB‐CT}^{DIBH\;\mathrm{mod}\;el}$ makes unachievable objective targets for $Plan_{FB‐CT}^{DIBH\;\mathrm{mod}\;el}$. The average OAR dose for ${\text{Prediction}}_{DIBH‐CT}^{FB\;\mathrm{mod}\;el}$ was higher than $Plan_{DIBH‐CT}^{\mathrm{manual}}$(*P* < 0.05 for all clinically relevant structures), and the estimated mean dose was slightly higher than the delivered one. It could be due to part of FB model predicted constraint conditions being too relaxed to limit the dose of OARs.

**Fig. 2:**
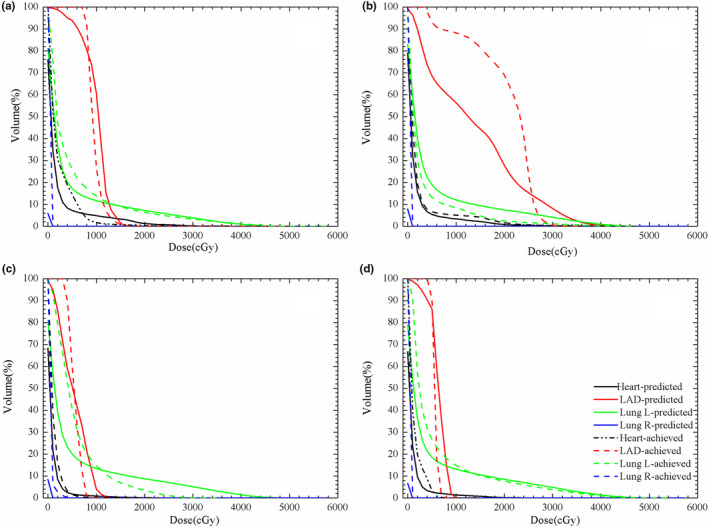
The dose volume histograms of the prediction (solid line) and the deliverable plan (dashed line) for (a) free breathing (FB) model work with FB‐computed tomography (CT); (b) deep inspiration breath hold (DIBH) model work with FB‐CT; (c) DIBH model work with DIBH‐CT; (d) FB model work with DIBH‐CT.

Figure 2 shows one representative example of four situations of the DVHs cross‐ validated by two KDE models. The dashed line represents the deliverable plan and the solid line represents the KDE prediction. The DVHs of the heart, LADCA, and left lung are shown in the black lines, red lines, and green lines, respectively.

All in all, in order to more easily and accurately guide the application of deep inhalation and breath holding in left‐breast cancer radiotherapy, we must quantify the protective capacity of DIBH for normal organs. As a method of evaluation, the KBP improves the consistency of all IMRT plans by minimizing the dissimilarity in plan quality due to the variation planner. The following operations can be used to guide the application of DIBH technology before radiotherapy: put FB‐CT and DIBH‐CT scans of the left‐breast patient into correspondence KDE model, then we can assess the degree of benefit from DIBH by comparison and analysis.

Evidently selecting the appropriate KBM model in this link is crucial. This work has demonstrated that one KDE model trained with one breath condition may not be suitable for all breath conditions. FB model cannot predict DIBH‐CT precisely because the establishment of FB model constraints is too loose for DIBH‐CT. This may cause plan quality degradation. Meanwhile, there are two problems when using the DIBH model work with FB‐CT: one is difficult for plan generation due to the harsh prediction constraints; the other is overtime for plan optimization. However, it is necessary to examine the effect of enlarging the sample size on the model presented in future research.

We consider this study innovative because it explains and validates the correlation between classification of the KDE‐based dose prediction model and breathing maneuvers during left‐sided whole‐breast irradiation after breast‐conserving surgery. It shows that classifying KDE dose prediction models according to respiratory patterns are indispensable, thus an optimal decision base for automatically making the radiation therapy plan of the marshalling station with computer is supplied. This research, while just a beginning, at least establishes some basic scientific facts that could prove useful in future studies on the automatic plan and related conditions.

## 5 CONCLUSION

The KDE model predicted DVHs and auto‐plan DVHs were not significantly different from the clinical DVHs when the appropriate model is used (FB model for FB plan, DIBH model for DIBH plan). The DIBH model should not be used for predicting FB treatments and vice versa. In view of the effective of the established model, the benefits of DIBH for a given patient can be quickly assessed before planning.

## CONFLICT OF INTEREST

None.
